# Phage Display Selection and In Silico Characterization of Peptides as Potential GroEL Modulators

**DOI:** 10.3390/pharmaceutics18010046

**Published:** 2025-12-30

**Authors:** Stefania Olla, Stella Garcia Colombarolli, Chiara Siguri, Davide Murrau, Alberto Vitali

**Affiliations:** 1 Institute for Genetic and Biomedical Research (IRGB), The National Research Council (CNR), Monserrato, 09042 Cagliari, Italy; chiara.siguri@irgb.cnr.it (C.S.); davide.murrau@cnr.it (D.M.); 2 René Rachou Institute, Oswaldo Cruz Foundation, Av. Augusto de Lima 1715, Belo Horizonte 30190-002, Minas Gerais, Brazil; stella.colombarolli@fiocruz.br; 3 Institute of Chemical Sciences and Technologies (SCITEC), The National Research Council (CNR), L.go F. Vito, 1, 00168 Roma, Italy

**Keywords:** peptide–protein docking, molecular dynamics, antibiotic resistance, chaperonin, peptide phage display

## Abstract

**Background**/**Objectives.** Antibiotic resistance is an escalating global health concern, highlighting the need for innovative antibacterial strategies beyond traditional drugs. GroEL, a highly conserved bacterial chaperonin essential for protein folding and stress tolerance, represents a promising but underexplored therapeutic target. This study aimed to identify short peptides capable of binding GroEL monomers and potentially altering their function, with the long-term goal of disrupting bacterial survival mechanisms. **Methods.** A phage display screening of a 12-mer peptide library was performed against purified GroEL monomers, yielding five candidate peptides (G1–G5). Their interactions with GroEL were analyzed through molecular docking and molecular dynamics simulations using three-dimensional GroEL structures (1MNF, 1XCK, 8S32). Stability of binding and interaction profiles were assessed through molecular dynamics-based analyses and MM/GBSA free energy calculations. **Results.** Peptides G4 and G5 displayed the most stable and energetically favorable interactions, with G4–8S32 showing the strongest binding (−116.68 kcal/mol). These peptides localized near inter-subunit interfaces, suggesting potential interference with GroEL oligomerization or allosteric transitions, which are critical for its biological function. **Conclusions.** Our findings demonstrate that short peptides can stably bind GroEL and potentially modulate its activity. Peptides G4 and G5 represent at our knowledge the first promising scaffolds for developing a novel class of peptide-based antibacterial agents targeting conserved chaperonin systems. This work introduces a new avenue that warrants further experimental validation.

## 1. Introduction

The alarming rise in antibiotic resistance has become one of the most pressing global public health challenges of the 21st century. Resistant bacterial strains continue to undermine the effectiveness of existing antimicrobial therapies, leading to increased morbidity, mortality, and healthcare costs worldwide [1,2]. Despite considerable efforts to develop new antibiotics, the drug pipeline remains limited, and resistance almost inevitably emerges following the introduction of new compounds. This scenario underscores the urgent need to explore alternative therapeutic strategies, particularly those targeting essential bacterial pathways and those involved in essential cellular processes. Among these, the bacterial chaperonin GroEL has recently gained attention as a potentially druggable protein essential for proteostasis and bacterial survival under stress conditions [3,4].

GroEL is a type I chaperonin found in nearly all eubacteria and works in concert with the co-chaperonin GroES to mediate the ATP-dependent folding of nascent or misfolded polypeptides [5]. Structurally, GroEL assembles into a tetradecameric barrel-like complex consisting of two stacked heptameric rings, each composed of 57 kDa subunits. Each subunit exhibits three domains: the apical domain (substrate binding), the intermediate domain (ATP binding and hydrolysis), and the equatorial domain (inter-ring contact and stabilization) [6]. The conformational dynamics of GroEL are central to its mechanism, as ATP binding, hydrolysis, and interaction with GroES coordinate the encapsulation and release of substrate proteins [7,8].

The GroEL–GroES system provides a protected environment that prevents aggregation and facilitates correct folding, often through multiple cycles of binding and release. Each encapsulation cycle, powered by ATP hydrolysis, provides the substrate with a ∼15 s window to fold. If the protein fails to reach its native state, it may undergo additional rounds of confinement and refolding [9,10]. Notably, during heat shock, increased levels of GroEL and GroES aid in managing thermally unstable proteins, and under extreme stress, GroEL may temporarily function as a “protein store” by slowing substrate release [11].

Targeting GroEL’s function or oligomeric assembly represents a rational strategy to impair bacterial viability. Disruption of its assembly into the functional tetradecamer could prevent proper protein folding, leading to proteotoxic stress and bacterial growth inhibition. Notably, GroEL is upregulated during heat shock and stress responses and is essential for viability in many pathogens, including *Escherichia coli*, *Mycobacterium tuberculosis*, and *Pseudomonas aeruginosa* [12]. Moreover, although certain organisms possess multiple isoforms of HSP60 or GroEL, at least one of them has consistently been found to be essential for survival in all microorganisms studied to date. Due to the highly conserved structure in several bacterial strains, GroEL/ES chaperonin systems could be a broad-spectrum antibiotic target. Because GroEL also shares significant structural and functional similarity with the eukaryotic chaperonin Hsp60, several studies have shown that eukaryotic cells may ‘misrecognize’ bacterial chaperonins as their own. This molecular mimicry can lead to internalization, receptor engagement, or immunomodulatory responses like those triggered by endogenous Hsp60, it further supports the relevance of targeting GroEL, as its conserved architecture not only underpins its essential role in bacterial proteostasis but also influences host–pathogen interactions. Diverse ways to develop direct inhibitors have been explored, especially by the group of Steven Johnson and collaborators who screened libraries of small organic molecules discovering many compounds able to inhibit GroEL activity with IC_50_ in the micromolar range [13,14,15,16]. Conversely, specific peptides able to inhibit chaperone machinery have not been developed to date. Some peptides have been used as probes to study GroEL mechanistic functioning [17,18], while some natural antimicrobial peptides have been characterized as unspecific ligands of GroEL [19,20]. In this context, the peptide–phage display technique provides a powerful platform for the discovery of peptide ligands with high affinity and specificity for protein targets [21,22]. By screening combinatorial peptide libraries against GroEL subunits in their monomeric form, it is possible to isolate peptides that bind specific structural motifs involved in inter-subunit interactions. Such peptides may function as molecular probes or inhibitors, preventing proper oligomerization or interfering with the chaperonin’s conformational cycle. Moreover, these ligands could provide valuable scaffolds for the development of innovative anti-infective agents, potentially acting synergistically with existing antibiotics, particularly against multidrug-resistant strains.

The aim of the present study is to identify peptide ligands that selectively bind GroEL monomers using phage display technology and to characterize their interaction through molecular modelling. By targeting monomeric subunits before they assemble into the functional complex, we seek to block GroEL activity at an early stage, thereby impairing chaperoning function and ultimately reducing bacterial survival. Molecular and molecular dynamics simulations offer detailed, atomistic insights into binding stability, flexibility, and interaction mechanisms—information that is often difficult to obtain experimentally due to the time and cost associated with such assays. While our computational and screening results highlight promising ligand candidates and a potentially novel anti-infective strategy, these findings require further experimental validation to confirm their biological relevance and assess their therapeutic potential. If corroborated, this approach may open new avenues for targeting conserved bacterial machinery, representing a paradigm shift in the design of next-generation antimicrobial agents.

## 2. Materials and Methods

### 2.1. Protein Preparation

GroEL protein was prepared based on the reported methodology [23] using a DH5a in which the *groEL* gene was inserted in pTrc99a (AmpR) under the control of the inducible Trc promoter (a kind gift from Steve Burston, Bristol University, Bristol, UK). This GroEL overproducing strain was grown in a LB medium. Cultured cells were induced with 1 mM Isopropyl β-d-1-thiogalactopyranoside (IPTG, Merck, Milan, Italy) at absorbance at 600 nm ~0.5–0.7 and allowed to grow overnight at 37 °C. Cells were then harvested by centrifugation at 5000 rpm and the supernatant removed. The pellet was then resuspended in Buffer A (50 mM Tris pH 7.5, 2 mM EDTA). Cells were lysed using a sonicator and the insoluble material removed by centrifuging at 20,000 rpm for 30 min. The soluble cell extract was then applied to a Fast-Flow Q-Sepharose (Merk, Milan, Italy approx. 40–50 mL of Q-Sepharose per 1 L culture) column pre-equilibrated in Buffer A and washed with the same buffer until the absorbance at 280 nm returns to zero. Proteins were eluted from the column using a linear gradient between Buffer A and 50% Buffer B (50 mM Tris pH 7.5 + 2 M NaCl). GroEL eluted at approximately 0.4 M NaCl (i.e., 20% Buffer B) in fractions 23–36. The Fast-Flow Q-Sepharose column washed with 100% Buffer B before re-equilibrating in Buffer A with 20% (*v*/*v*) HPLC-grade methanol (the methanol removes bound impurities). The collected GroEL fractions checked after EF-SDS, were then re-applied to the FF Q-Sepharose column and washed with one column volume of Buffer A containing 20% methanol. The GroEL was then eluted from the column using a linear gradient between Buffer A containing 20% methanol and 50% Buffer B containing 20% methanol. As above, the GroEL elutes at approximately 0.4 M NaCl (Figure S1). The concentration of GroEL was determined spectrophotometrically by measuring an absorbance spectrum from 320 nm to 250 nm and subtracting the absorbance at 300 nm from the absorbance at 280 nm to get a true reading of the absorbance at 280 nm (GroEL is large enough to scatter a small proportion of light at 300 nm). A molar extinction coefficient of 9600 M^−1^ cm^−1^ (at 280 nm) for GroEL subunits was used.

A commercial preparation of GroEL from Sigma-Aldrich (Cat. N.C7688, Milan, Italy) was also used when necessary. The purified monomers were used as bait in the following selection of peptides by phage display biopannings.

### 2.2. Selection of GroEL-Specific Binding Peptides via Phage Display

Phage display was employed to identify peptide ligands with specific affinity toward the molecular chaperonin GroEL. Commercial Ph.D.™-12 Phage Display Peptide Library Kit (New England Biolabs, Ipswich, MA, USA, Cat. #E8110S) was used, containing M13 filamentous phage particles displaying random 12-mer peptides fused to the pIII coat protein.

High-binding polystyrene 96-well plates were coated with 100 µL of recombinant purified GroEL (0.25 µg/µL in 0.05 M carbonate–bicarbonate buffer) and incubated overnight at 4 °C under gentle agitation. After removal of the coating solution, wells were blocked with PBS containing 5% bovine serum albumin (BSA) for 1 h at 4 °C, followed by six rapid washes with TBST (TBS + 0.1% Tween-20).

Approximately 10^11^ phage particles were incubated in GroEL-coated wells for 1 h at room temperature with gentle agitation. Non-binding phages were removed through ten washes with TBST. Bound phages were eluted using 0.2 M Glycine-HCl (pH 2.2) supplemented with 1 mg/mL bovine serum albumin from Sigma-Aldrich (St. Louis, MO, USA), incubated for 8 min, and neutralized with 15 µL of 1 M Tris-HCl (pH 9.1).

Eluted phages were tittered via infection of *E. coli* strain ER2738 and plated on LB/IPTG/X-Gal agar. Blue plaques were counted after overnight incubation at 37 °C. Individual clones were isolated, amplified in ER2738 overnight cultures, and subjected to plasmid extraction using QIAprep spin miniprep kit from QIAGEN (Venlo, The Netherlands). DNA concentration was quantified using a Nanodrop 2000 spectrophotometer from Thermo Scientific (Waltham, MA, USA), and sequences were determined via Sanger sequencing. Three independent rounds of selection rounds were subsequently performed to increase the specificity and/or diversity of potential GroEL-binding candidates. Across all these attempts, no significant differences in phage titers were observed compared with the first round, which consistently yielded a limited number of phage-infected colonies per experiment. All recovered colonies were subjected to sequencing analysis using the −96 primer provided with the Ph.D.™-12 Phage Display Peptide Library Kit (New England Biolabs, Cat. (#E8110S). Sequencing was performed by BMR Genomics, and the obtained sequences were analysed using MEGA (MEGA software 12.1).

### 2.3. In Silico Studies

#### 2.3.1. Protein Structure Preparation

Three protein structures were selected from the Protein Data Bank (RCSB PDB) [24], ensuring that they did not contain any mutations in the amino acid sequences. Specifically, we selected two X-ray crystallography-derived structures—1MNF [25], complexed with an oligopeptide, and 1XCK [26], in its apo form—and one structure determined by cryo-electron microscopy, 8S32, bound to a GroTAC peptide [27].

For each structure, a single monomer was retained. The protein models were prepared using the Protein Preparation Wizard tool (Schrödinger Release 2024-3: Protein Preparation Wizard) [28]. During preparation, water molecules were removed, missing side chains and loops were rebuilt, when necessary, bond orders were assigned using the Chemical Component Dictionary (CCD) database, and hydrogen atoms were added and optimized. Protonation and hydrogen bonding networks were further refined with PROPKA-based optimization [29], and heavy atoms were energy-minimized until convergence to a root-mean-square deviation (RMSD) of 0.30 Å.

#### 2.3.2. Docking with AutoDock CrankPep (ADCP)

Affinity maps for docking were generated using AutoGridFR (AGFR ADFRsuite 1.0) with default parameters, defining a grid box that encompassed the entire structure of the target protein. AGFR produced a configuration file (.trg), which was used as the receptor input in the subsequent docking step performed with AutoDock CrankPep (ADCP ADFRsuite 1.0) [30]. Docking was carried out using ADCP’s default settings, which include 3 million scoring function evaluations per amino acid residue of the peptide during each Monte Carlo search cycle. The previously prepared three-dimensional structures of the peptides were used as ligands. For each peptide–protein complex, 300 binding poses were generated and then clustered to identify the most representative binding configurations.

#### 2.3.3. Molecular Dynamics

The best results, undergoing molecular dynamics (MD) studies, were based on the optimal poses obtained from docking programs.

MD studies were conducted using Desmond simulation package of Schrödinger LLC (New York, NY, USA) [31], (GPU implementation) on NVDIA graphic card (RTX A5000), and the TIP3P solvent model [32] were utilized in conjunction with the OPLS4 force field [33]. To set up the system, each complex was positioned in an orthorhombic water box with a 12.0 Å extension, Sodium and chloride ions were added randomly in the water phase to neutralize the systems and reach the experimental salt concentration of 0.150 M NaCl. The simulations extended for 500 ns, with trajectories recorded at intervals of 100 ps within the NPT ensemble. The temperature (300.0 K) and pressure (1.01325 bar) were consistently maintained using the Langevin thermostat and barostat [34] methods, respectively. Other parameters were kept at their default values. The analysis was performed using the Simulation Interaction Diagram tool integrated into Desmond, and the obtained trajectories were further subjected to Molecular Mechanics Generalized Born Surface Area (MM/GBSA) analysis using the thermal_mmgbsa.py script. Additionally, trajectory frames were subjected to clustering based on ligand RMSD using the trj_cluster.py script [35]. Output files were generated for the five most populated clusters in each MD experiment.

## 3. Results

### 3.1. Groel Purification

The purification protocol described in the experimental section allowed to obtain high purity monomers of GroEL (Figure S1). These fractions were hence used in the following phage display trials as baits.

### 3.2. Phage Display

As a result of the biopanning selection, five unique peptide sequences were identified, showing potential binding affinity to GroEL: HNMHKGDNYYHG (G1), AVAPGLIKYGTR (G2), METRPVAPHEFR (G3), SHNSTDVHLLLR (G4), TPLWQDNRALGS (G5). Further selection attempts were made, but no significant differences in phage titers were observed compared with the first round. Table 1 shows some chemical and physical characteristics of the five peptides relating to their ability to interact with biological membranes.

### 3.3. Docking Studies

The five peptides (G1-5) obtained through phage display were docked onto three-dimensional (3D) structures of GroEL: 1MNF (X-ray structure bound to an oligopeptide), 1XCK (apo X-ray structure), and 8S32 (cryo-EM structure bound to a GroTAC peptide). All structures were prepared for docking using standard protein preparation protocols. The docking results are summarized in Table 2.

For each peptide–protein complex, 300 poses were generated and clustered based on spatial similarity. Pose selection for subsequent molecular dynamics simulations was based on three main criteria: cluster size, docking score, and binding mode. Based on these parameters, the least promising pose from one of the three structures was discarded for each peptide. Therefore, for every peptide, the two most promising docking poses—each from a different 3D structure were retained for further analysis. Specifically, for peptides G1, G2, G4, and G5, the poses obtained with structures 8S32 and 1MNF were retained, while for G3, the most promising ones were those derived from 8S32 and 1XCK.

### 3.4. Molecular Dynamics

Molecular dynamics (MD) simulations were performed for all five selected peptides in complex with the two GroEL structures that had shown the best docking results. In total, ten simulations were carried out, each lasting 500 ns. These simulations aimed to assess the stability of the peptide–protein complexes over time and to provide insights into their dynamic behavior under near-physiological conditions.

**Figure 1:**
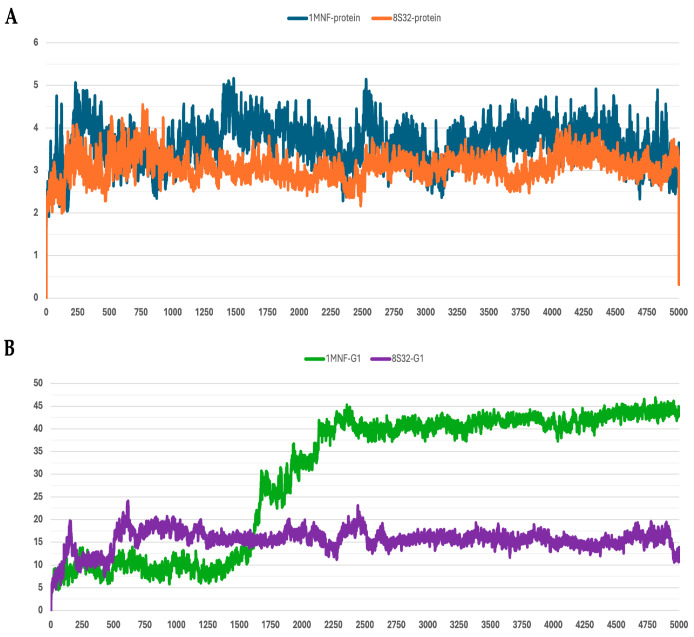
RMSD profiles protein backbones and peptide G1 during molecular dynamics simulations. The *x*-axis reports the simulation frames, (one frame every 0.1 ns), while the *y*-axis shows the RMSD values in Å. (**A**) RMSD profiles of GroEL models based on the 8S32 (protein in orange) and 1MNF (protein in blue) structures. (**B**) RMSD profiles of peptide G1 in complex with GroEL structures 8S32 (peptide in purple) and 1MNF (peptide in green).

**Figure 2:**
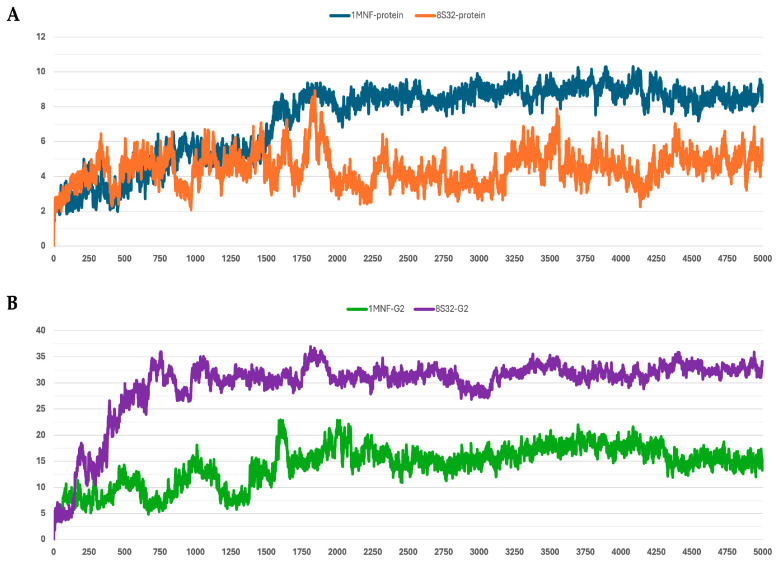
RMSD profiles protein backbones and peptide G2 during molecular dynamics simulations. The *x*-axis reports the simulation frames, (one frame every 0.1 ns), while the *y*-axis shows the RMSD values in Å. (**A**) RMSD profiles of GroEL models based on the 8S32 (protein in orange) and 1MNF (protein in blue) structures. (**B**) RMSD profiles of peptide G2 in complex with GroEL structures 8S32 (peptide in purple) and 1MNF (peptide in green).

**Figure 3:**
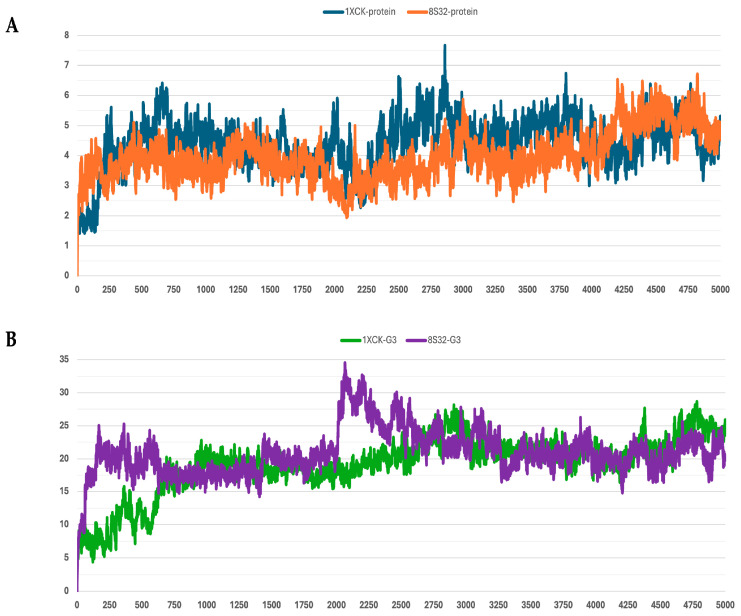
RMSD profiles protein backbones and peptide G3 during molecular dynamics simulations. The *x*-axis reports the simulation frames, (one frame every 0.1 ns), while the *y*-axis shows the RMSD values in Å. (**A**) RMSD profiles of GroEL models based on the 8S32 (protein in orange) and 1XCK (protein in blue) structures. (**B**) RMSD profiles of peptide G3 in complex with GroEL structures 8S32 (peptide in purple) and 1XCK (peptide in green).

**Figure 4:**
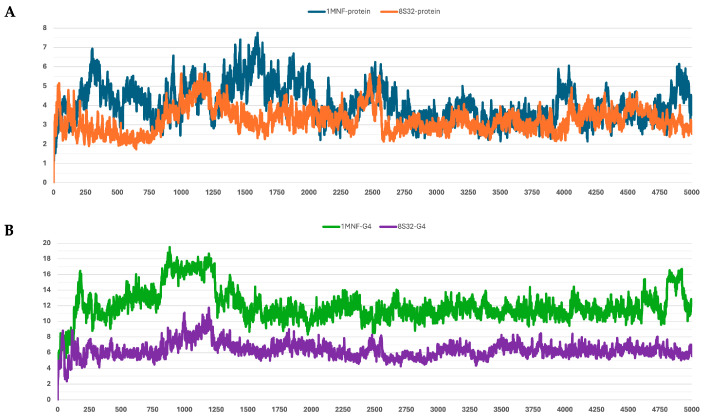
RMSD profiles protein backbones and peptide G4 during molecular dynamics simulations. The *x*-axis reports the simulation frames, (one frame every 0.1 ns), while the *y*-axis shows the RMSD values in Å. (**A**) RMSD profiles of GroEL models based on the 8S32 (protein in orange) and 1MNF (protein in blue) structures. (**B**) RMSD profiles of peptide G4 in complex with GroEL structures 8S32 (peptide in purple) and 1MNF (peptide in green).

**Figure 5:**
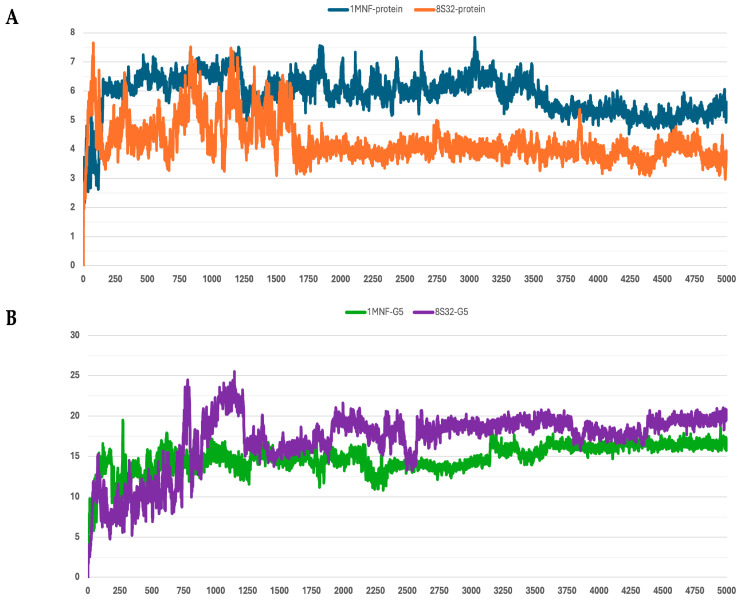
RMSD profiles protein backbones and peptide G5 during molecular dynamics simulations. The *x*-axis reports the simulation frames, (one frame every 0.1 ns), while the *y*-axis shows the RMSD values in Å. (**A**) RMSD profiles of GroEL models based on the 8S32 (protein in orange) and 1MNF (protein in blue) structures. (**B**) RMSD profiles of peptide G5 in complex with GroEL structures 8S32 (peptide in purple) and 1MNF (peptide in green).

The stability of the peptide–protein complexes during the simulations was mainly evaluated by analyzing the RMSD of backbone atoms. The RMSD profiles of all trajectories are shown in Figure 1, Figure 2, Figure 3, Figure 4 and Figure 5, and the corresponding values are reported in Table 3.

Among the analyzed systems, the most stable complex was observed for peptide G4 bound to the GroEL structure 8S32, with RMSD values of 3.26 ± 0.66 Å and 6.33 ± 1.0 Å for the protein and the ligand, respectively. This stability is also clearly reflected in Figure 4A,B. Peptide G5 also exhibited good stability when bound to both GroEL structures (1MNF and 8S32 see Figure 5B). In the case of the G5–8S32 complex, the peptide showed some instability during the first 100 ns, associated with a binding mode of rearrangement, after which the system reached a more stable conformation.

Subsequently, the MM/GBSA binding free energy was calculated for each simulation, and the results are presented in Table 4. From an energetic perspective, the most stable complex was that formed by peptide G4 bound to structure 8S32, with a binding free energy of −116.68 kcal/mol. In contrast, the complex formed by peptide G3 with the same structure was the least energetically favored, with a binding free energy of −37.88 kcal/mol. Notably, peptide G5 exhibited favorable binding free energies with both structures, 8S32 and 1MNF, with values of −58.07 kcal/mol and −57.48 kcal/mol, respectively.

**Figure 6:**
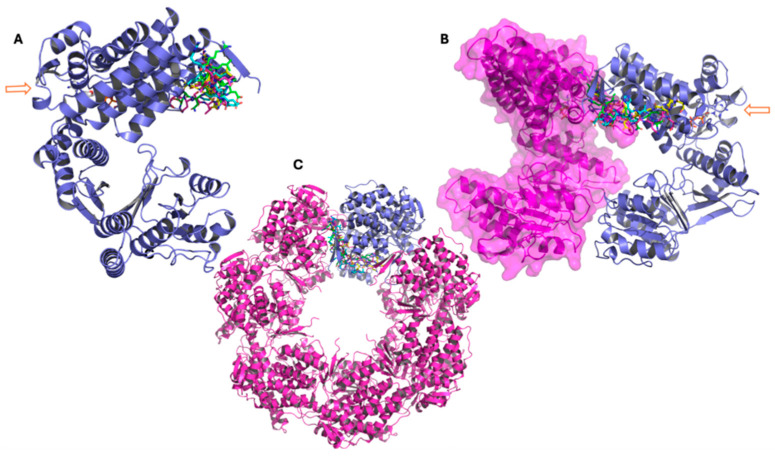
Representative conformations of peptide G4 from molecular dynamics simulations performed with the GroEL 3D structure 8S32, showing the main clusters derived from trajectory analysis. (**A**–**C**): The three panels show the GroEL complex at different structural levels. In all representations, the peptide conformation from docking is shown in cyan, while the three main clusters obtained from molecular dynamics simulations are represented in green (cluster 1), magenta (cluster 2), and yellow (cluster 3). (**A**) GroEL monomer (violet), with the arrow indicating the ATP molecule and the atypical ATP-binding site. (**B**) Dimeric GroEL structure showing the second monomer in magenta with surface representation. The arrow indicates the ATP molecule and the atypical ATP-binding site. (**C**) Heptameric GroEL complex (magenta), highlighting the monomer used for docking and molecular dynamics studies in violet.

Binding-mode and clustering analyses were carried out for each peptide in the complex with the GroEL structure that produced the most favorable free-energy outcome (Table 4). For peptide G4–8S32, the docking pose (in cyan) and the three most populated clusters throughout the 500 ns simulation converge on the same binding pocket (Figure 6A). The peptide remains firmly anchored, demonstrating persistent interaction during the dynamic simulation. In Figure 6B, G4 is shown engaging the interface between two GroEL monomers, suggesting the potential to interfere with oligomer formation; the corresponding structural position is further illustrated in Figure 6C. Although the arrow in panel B highlights proximity to the ATP-binding site, the peptide does not appear to directly perturb the catalytic region.

**Figure 7:**
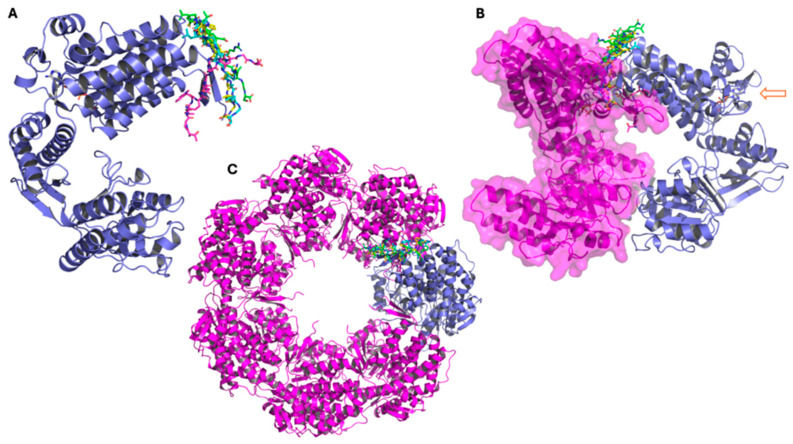
Representative conformations of peptide G5 from molecular dynamics simulations performed with the GroEL 3D structure 8S32, showing the main clusters derived from trajectory analysis. (**A**–**C**): The three panels show the GroEL complex at different structural levels. In all representations, the peptide conformation from docking is shown in cyan, while the three main clusters obtained from molecular dynamics simulations are represented in green (cluster 1), magenta (cluster 2), and yellow (cluster 3). (**A**) GroEL monomer (violet), with the peptide conformation from docking (in cyan), and cluster1 (in green), cluster 2 (in magenta), and cluster 3 (in yellow). (**B**) Dimeric GroEL structure showing the second monomer in magenta with surface representation. The arrow indicates the ATP molecule and the atypical ATP-binding site. (**C**) Heptameric GroEL complex (magenta), highlighting the monomer used for docking and molecular dynamics studies in violet.

Peptide G5–8S32 displays a similar binding behavior to G4: its docking pose and dominant clusters remain within the same pocket during the simulation and are positioned adjacent to inter-subunit regions (but not directly at the ATP-binding region) (Figure 7). This suggests that G4 and G5 could exhibit a dual mode of modulation, potentially interfering with inter-subunit contacts while also exerting subtle allosteric effects on the catalytic region.

Peptide G2–8S32 also retains binding stability, with docking and cluster conformations remaining within the same binding site throughout the simulation (Figure S3). Unlike G4 and G5, G2’s binding site lies further from the ATP-binding region, hinting at a mechanism of modulation through conformational perturbation rather than direct catalysis interference.

By contrast, peptides G1–1MNF and G3–1XCK exhibit different binding behaviors: their dominant clusters are localized in regions distinct from the inter-monomer interfaces or ATP-proximal pockets (see Supplementary Figures S2 and S4). Notably, G1 and G3 show greater structural fluctuations and less converged binding poses compared to G4, G5 and G2. Taken together, these data support the hypothesis that the isolated peptides may modulate GroEL function via multiple mechanisms: for G4 and G5 by interfering with inter-monomer contacts and indirectly affecting the ATPase cycle, for G2 by inducing structural modulation away from the catalytic core, and for G1/G3 by engaging alternative interaction sites with potentially different functional consequences.

## 4. Discussion

The development of new antibacterial molecules that act on pathways distinct from those targeted by conventional drugs is crucial for addressing the escalating challenge of antibiotic resistance. In this study, we investigated the bacterial chaperonin GroEL (HSP60) a central component of proteostasis and stress response, evaluating its suitability as a drug target. To identify potential ligands capable of modulating GroEL function, we used peptide–phage display to isolate short peptides binding GroEL monomers, with the aim of interacting with GroEL oligomerization or its ATP-driven conformational cycle. A single round of biopanning yielded five peptide sequences (G1–G5) isolated from their respective clones. The fact that no further enrichment of colonies was observed in subsequent cycles may be due to the high homogeneity of the target consisting of purified GroEL monomers. This observation prompted a detailed computational characterization through molecular docking and molecular dynamics (MD) simulations, enabling the identification of preferred binding regions and identifying potential surface hotspots. The results suggest that these peptides might interfere with GroEL oligomerization, destabilize its structural integrity, or modulate allosteric transitions.

Docking analyses performed on three GroEL structures (1MNF, 1XCK, 8S32), revealed comparable binding energies across all peptides, with docking scores ranging from −17 to −22 kcal/mol. Peptides G1, G4 and G5 ranked among the most favorable. MD simulations confirmed that most complexes reached equilibrium after an initial relaxation phase, with G4–8S32 and G5–8S32 displaying the lowest RMSD fluctuations. Consistently, MM/GBSA calculations identified the G4–8S32 complex as the stronger binder (−116.68 kcal/mol), followed by G5–8S32 and G5–1MNF, both showing intermediate yet significant binding free energies (around −58 kcal/mol).

Among the analyzed complexes, G4-8S32 and G5-8S32 were found to bind within surface cavities near inter-subunit or domain-interface regions (Figure 6 and Figure 7). These binding sites align with a mechanism involving interference with GroEL assembly or allosteric regulation, rather than direct competition with substrate or ATP binding sites. Binding at subunit interfaces or flexible hinge regions could inhibit or disrupt the assembly of the tetradecameric GroEL complex or perturb ATP-dependent conformational transitions [5,7], ultimately compromising the chaperonin’s folding activity. This may be an alternative mechanism respect with those described is consistent with mechanisms described for synthetic GroEL modulators [13,14].

Our findings extend this concept by demonstrating that short peptides selected via phage display can exhibit comparable binding behavior in silico. To our knowledge, peptides directly interacting and modulating GroEL have not been previously reported. However, well-known antimicrobial peptides of insect origin such as Pyrrhocoricin interact with other chaperones like DnaK, interfering with the DnaK/DnaJ/GrpE cycle required for the proper folding of bacterial proteins. These peptides do not seem to have GroEL as a specific protein target [19,36]. Pyrrhocoricin was the first antibacterial peptide shown to inhibit an essential chaperone (DnaK) blocking both its ATPase activity and its folding function. These findings inspired the concept—further supported by our work—of using peptides as modulators of bacterial chaperones proteins (Hsp60/Hsp70) as an alternative to classical antibiotic strategies. As mentioned above, the phage display-derived peptides may act through a mechanism involving inhibition or misregulation of the GroEL complex formation, distinguishing them from Pyrrhicoricin.

Beyond binding affinity, biological activity depends on intracellular accessibility. A key determinant of a peptide’s activity within cells is its capacity to traverse cellular membranes. None of the peptides G1–G5 exhibit pronounced membrane activity (Table 1) according to classical parameters (GRAVY > 1 and amphipathicity > 0.5), a consideration that must be taken into account when planning in vitro investigations. Another crucial aspect is specificity. Chaperonins are highly conserved, and cross-recognition between bacterial and eukaryotic Hsp60 homologs has been documented. Although our predicted binding sites appear to be GroEL-specific, potential interactions with human or mitochondrial Hsp60 cannot be excluded and should be assessed experimentally.

Overall, our results suggest that peptides G4 and G5 exhibit the highest intrinsic affinity for GroEL and may act as modulators of its structural dynamics. In contrast, other peptides such as G3 showed moderate or weak stability, particularly when docked to the apo form of GroEL (1XCK), highlighting possible conformational selectivity in peptide recognition. Such selectivity may be advantageous for designing compounds that specifically interfere with distinct allosteric states of GroEL rather than permanently blocking its catalytic activity. Future studies should include cellular uptake assays, analyses of GroEL oligomerization, and selectivity tests against eukaryotic Hsp60 to comprehensively assess the therapeutic potential of these peptides.

## 5. Conclusions

Our data demonstrate that small peptides can form stable—albeit potentially transient—interactions with GroEL monomers. Among the identified ligands, G4 emerged as promising scaffolds for the rational design of peptide-based GroEL modulators. Further experimental validation will be required to confirm their binding affinities and to determine whether these peptides can effectively inhibit GroEL ATPase activity or impair substrate refolding in vitro.

## Figures and Tables

**Table 1:** Physico-chemical properties of phage display-derived peptides. Data retrieved from APD6 (https://aps.unmc.edu/prediction) and from * https://perseucpp.ufv.br/run_page. URLs accessed on 10 December 2025.

Peptide	Net Charge	GRAVY	WW Whole-Residue Hydrophobicity	Amphipaticity Index	H-Moment	Predicted Membrane Penetration *
G1	+0.75	−2.125	1.48	0.45	0.85	low
G2	+2	0.275	1.01	0.27	1.24	low
G3	+0.25	−1.03	5.75	0.45	0.76	low
G4	+0.5	−0.38	1.59	0.36	0.89	low
G5	0	−0.833	0.97	0.36	0.94	low

**Table 2:** Docking results of the five phage display-derived peptides on the three-dimensional structures of GroEL: 8S32, 1MNF and 1XCK.

Peptide	Results Docking 8S32 Kcal/mol	Results Docking 1MNF Kcal/mol	Results Docking 1XCK Kcal/mol
G1	−22.1	−22.2	−21.6
G2	−20.8	−19.5	−20.4
G3	−19.9	−17.1	−17.8
G4	−20.5	−19.8	−20.7
G5	−19.6	−19.5	−18.7

**Table 3:** Average RMSD (Å) values ± standard deviation for the five peptides (G1–G5) and their corresponding protein backbones during molecular dynamics simulations with GroEL structures 8S32, 1MNF, and 1XCK.

Peptide	RMSD 8S32	RMSD 1MNF	RMSD 1XCK
G1 peptide	15.58 ± 2.33	29.92 ± 14.74	
protein	3.09 ± 0.32	3.64 ± 0.49	
G2 peptide	29.72 ± 5.99	14.26 ± 3.85	
protein	4.47 ± 0.98	7.35 ± 2.12	
G3 peptide	20.91 ± 3.49		19.19 ± 4.37
protein	3.98 ± 0.79		4.44 ± 0.84
G4 peptide	6.33 ± 1.0	12.04 ± 2.04	
protein	3.26 ± 0.66	4.08 ± 0.97	
G5 peptide	17.09 ± 3.57	14.82 ± 1.72	
protein	4.27 ± 0.71	5.88 ± 0.70	

**Table 4:** MM/GBSA binding free energies from molecular dynamics simulations of peptides G1–G5 with GroEL structures 8S32, 1MNF and 1XCK.

Peptide	MMGBSA 8S32 Kcal/mol	MMGBSA 1MNF Kcal/mol	MMGBSA 1XCK Kcal/mol
G1	−45.08 ± 10.85	−48.10 ± 14.78	
G2	−59.42 ± 18.35	−41.65 ± 11.13	
G3	−37.88 ± 10.62		−53.29 ± 11.71
G4	−116.68 ± 15.85	−51.32 ± 14.48	
G5	−58.07 ± 24.04	−57.48 ± 19.19	

## Data Availability

All the data and simulations supporting the findings of this study are available from the corresponding author upon request.

## Supplementary Materials

The following supporting information can be downloaded at: https://www.mdpi.com/article/10.3390/pharmaceutics18010046/s1, Figure S1: Purification scheme of GroEL from *E. coli*; Figure S2: Representative conformations of peptide G1 from molecular dynamics simulations performed with the GroEL 3D structure 1MNF, showing the main clusters derived from trajectory analysis; Figure S3: Representative conformations of peptide G2 from molecular dynamics simulations performed with the GroEL 3D structure 8S32, showing the main clusters derived from trajectory analysis. Figure S4: Representative conformations of peptide G3 from molecular dynamics simulations performed with the GroEL 3D structure 1XCK, showing the main clusters derived from trajectory analysis.

## Abbreviations

The following abbreviations are used in this manuscript:

RMSD	Root Mean Square Deviation
IPTG	Isopropyl β-D-1-thiogalactopyranoside
EDTA	Ethylenediaminetetraacetic acid
EF-SDS	Sodium dodecyl sulfate–polyacrylamide gel electrophoresis
TBS	Tris-Buffered Saline
MMGBSA	Molecular Mechanics Generalized Born Surface Area
