# Supplementary material for: Phage Display Selection and In Silico Characterization of Peptides as Potential GroEL Modulators
Source: Pharmaceutics. 2025 Dec 30;18(1):46. doi: 10.3390/pharmaceutics18010046 (PMC12844741; doi:10.3390/pharmaceutics18010046)
Supplement: Supplementary file 1 [file pharmaceutics-18-00046-s001.zip › pharmaceutics-4009786-supplementary.pdf]

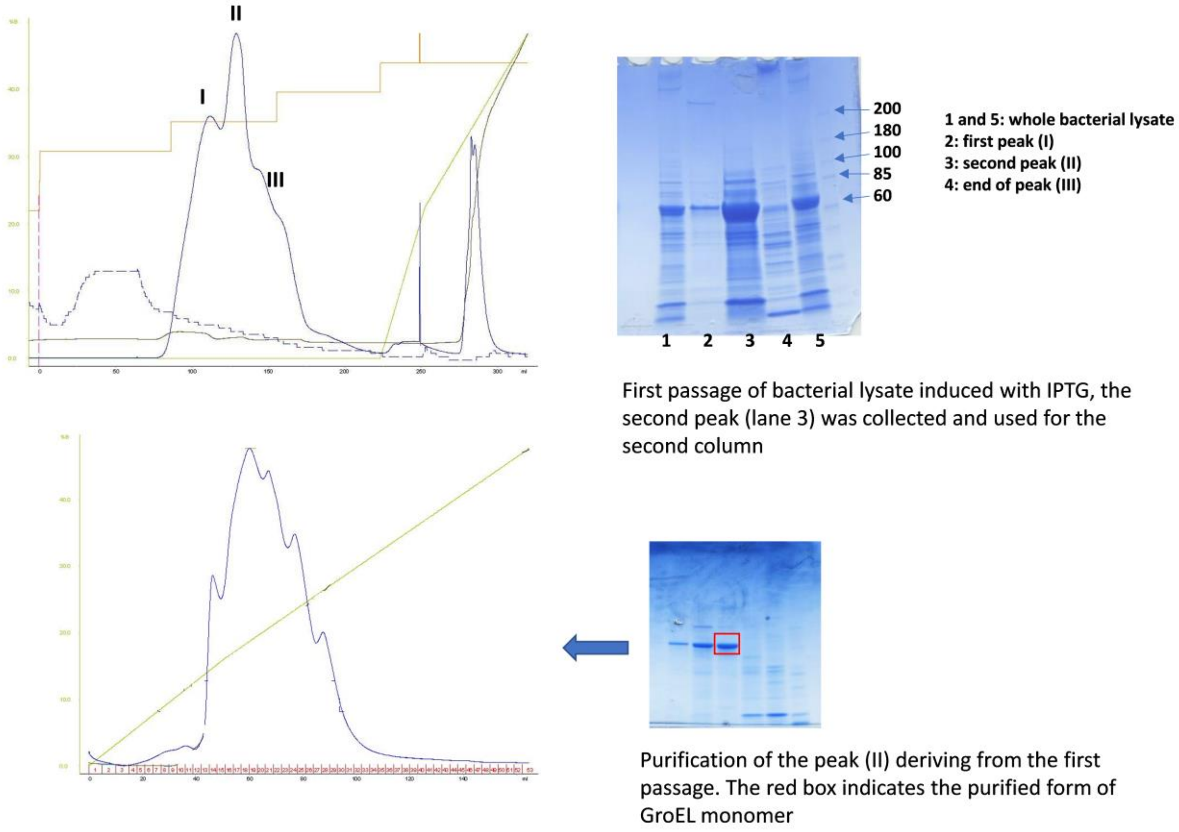

Figure S1. Purification scheme of GroEL from *E. coli*. A) First DEAE step, the peak in the frame was collected and further submitted to another DEAE step B) in which the fractions 23-36 were collected as containing the purest form of GroEL.

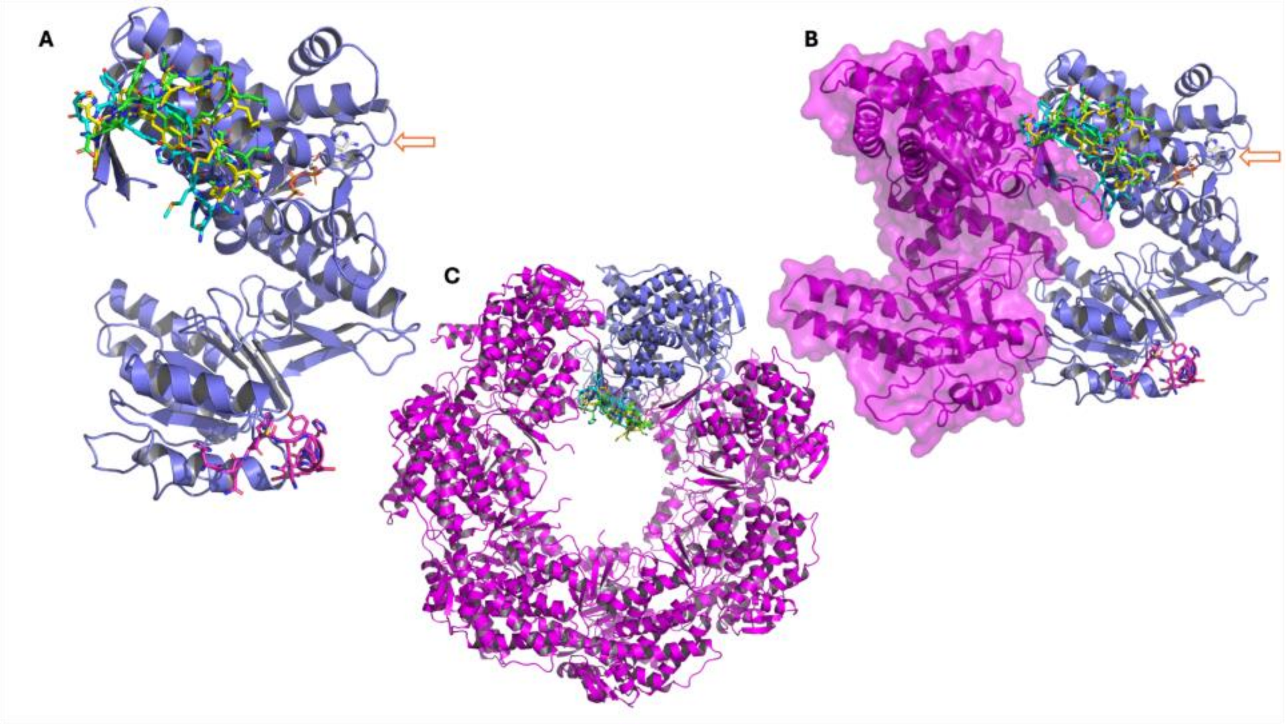

Figure S2. Representative conformations of peptide G1 from molecular dynamics simulations performed with the GroEL 3D structure 1MNF, showing the main clusters derived from trajectory analysis. A–C: The three panels show the GroEL complex at different structural levels. In all representations, the peptide conformation from docking is shown in cyan, while the three main clusters obtained from molecular dynamics simulations are represented in green (cluster 1), magenta (cluster 2), and yellow (cluster 3). (**a**) GroEL monomer (violet), with the arrow indicating the ATP molecule and the atypical ATP-binding site. (**b**) Dimeric GroEL structure showing the second monomer in magenta with surface representation. The arrow indicates the ATP molecule and the atypical ATP-binding site. (**c**) Heptameric GroEL complex (magenta), highlighting the monomer used for docking and molecular dynamics studies in violet.

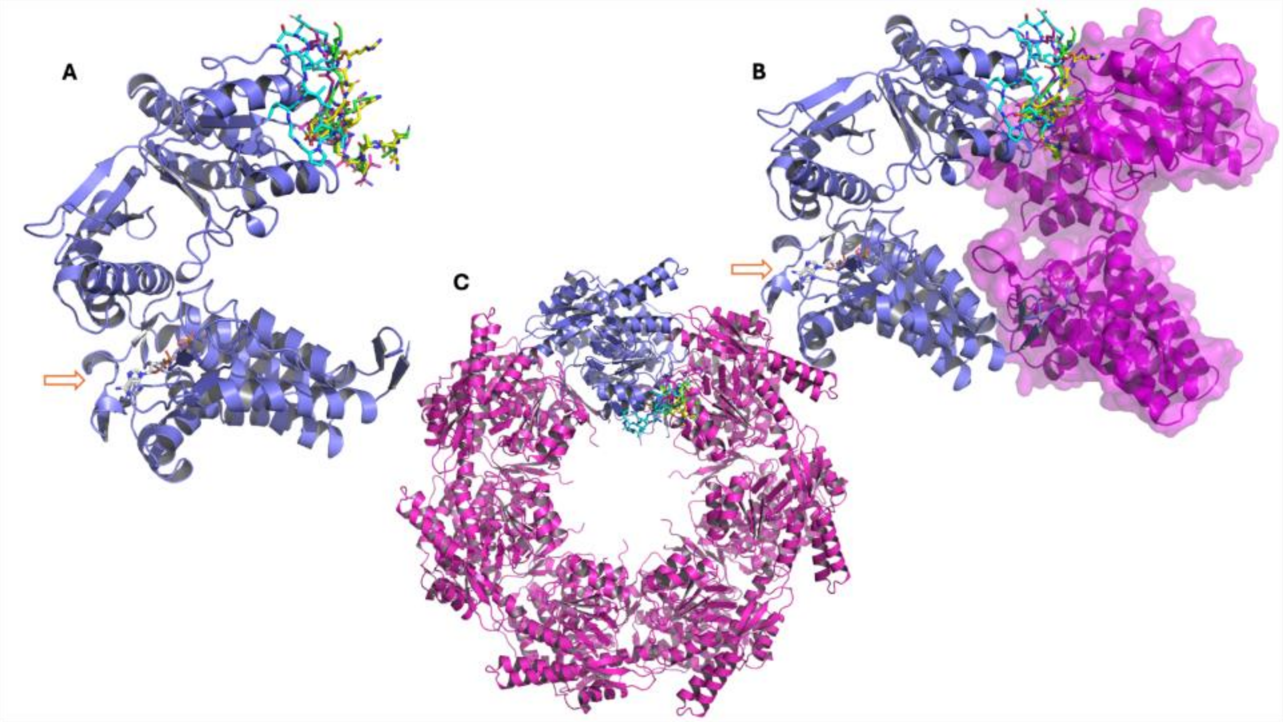

Figure S3. Representative conformations of peptide G2 from molecular dynamics simulations performed with the GroEL 3D structure 8S32, showing the main clusters derived from trajectory analysis. A–C: The three panels show the GroEL complex at different structural levels. In all representations, the peptide conformation from docking is shown in violet, while the three main clusters obtained from molecular dynamics simulations are represented in green (cluster 1), magenta (cluster 2), and yellow (cluster 3). (**a**) GroEL monomer (violet), with the arrow indicating the ATP molecule and the atypical ATP-binding site. (**b**) Dimeric GroEL structure showing the second monomer in magenta with surface representation. The arrow indicates the ATP molecule and the atypical ATP-binding site. (**c**) Heptameric GroEL complex (magenta), highlighting the monomer used for docking and molecular dynamics studies in violet.

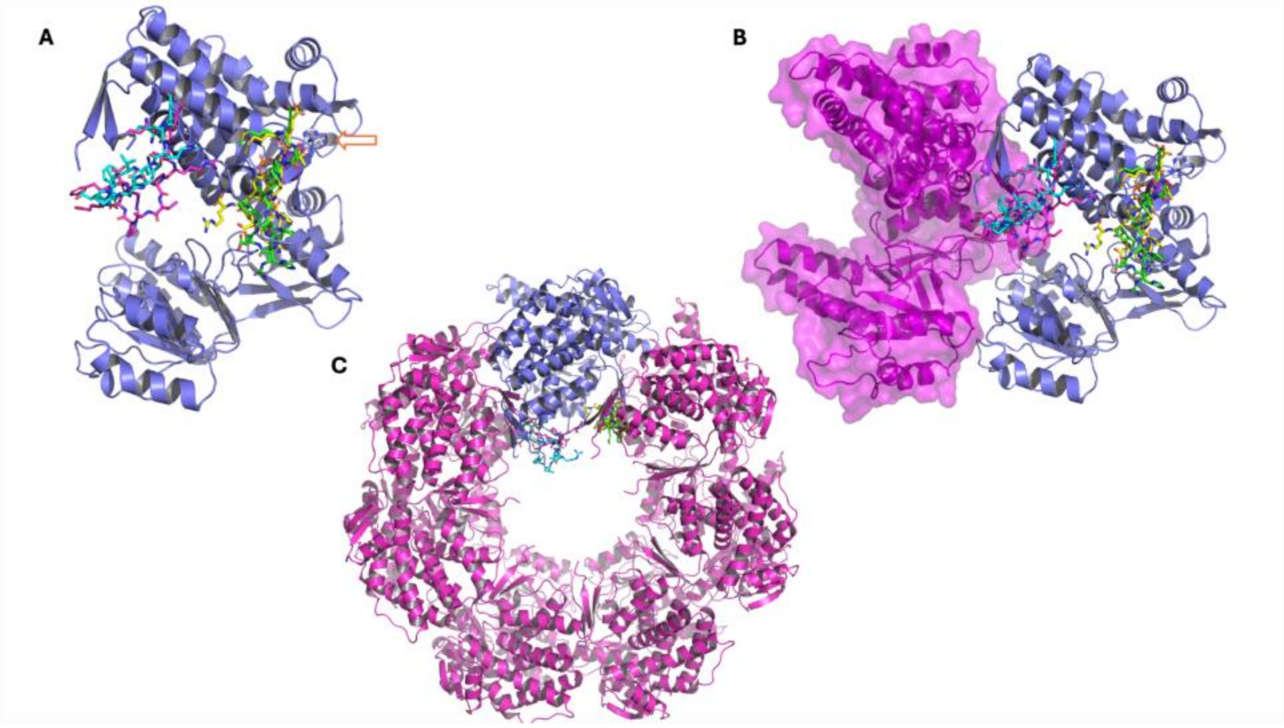

Figure S4. Representative conformations of peptide G3 from molecular dynamics simulations performed with the GroEL 3D structure 1XCK, showing the main clusters derived from trajectory analysis. A–C: The three panels show the GroEL complex at different structural levels. In all representations, the peptide conformation from docking is shown in cyan, while the three main clusters obtained from molecular dynamics simulations are represented in green (cluster 1), magenta (cluster 2), and yellow (cluster 3). (**a**) GroEL monomer (violet), with the arrow indicating the ATP molecule and the atypical ATP-binding site. (**b**) Dimeric GroEL structure showing the second monomer in magenta with surface representation. (**c**) Heptameric GroEL complex (magenta), highlighting in violet the monomer used for docking and molecular dynamics studies.
